# Hybrid endovascular and microsurgical treatment for a large mesencephalic-cerebellar hemangioblastoma

**DOI:** 10.3171/2019.10.FocusVid.19287

**Published:** 2019-10-01

**Authors:** Jianping Song, Peiliang Li, Yanlong Tian, Xiaochun Zhao, Xiaowen Wang, Wei Zhu

**Affiliations:** 1 Department of Neurosurgery, Huashan Hospital, Shanghai Medical College, Fudan University, Shanghai, China; and; 2 Department of Neurosurgery, Barrow Neurological Institute, St. Joseph’s Hospital and Medical Center, Phoenix, Arizona

**Keywords:** brainstem, hemangioblastoma, hybrid treatment, video

## Abstract

The large intracranial hemangioblastoma is a top surgical challenge due to its nature of invading brain parenchyma, tight adherence to the pia, and rich blood supply from numerous pial vasculatures and arteries in the proximity. If the brainstem is involved in the lesion, the surgery will be more dangerous because of potential brainstem impingement. In this illustrative video, we present a case of a 54-year-old male with a large hemangioblastoma at the mesencephalic-cerebellar region, which was successfully treated by hybrid endovascular embolization and microsurgery via an occipital interhemispheric transtentorial approach with minimal intraoperative blood loss and a favorable outcome.

The video can be found here: https://youtu.be/pJqFhY_Zhv0.

## Transcript

**0:20** This video illustrates a hybrid endovascular and microsurgical treatment for a large mesencephalic-cerebellar hemangioblastoma via an occipital interhemispheric transtentorial approach.

**0:32** A 54-year-old male presented with dizziness for the past 2 months. The symptom progressed gradually and he was unable to walk steadily in the recent 2 weeks with an ataxic gait. The laboratory test did not find any abnormality.

**0:46** The initial computed tomography scan showed a heterogeneous lesion with low density in the left cerebellum. From magnetic resonance images, an enhanced lesion with a low signal on T1, high signal on T2, which located at the mesencephalic-cerebellar region accompanied by the surrounding edema involving the dentate nucleus and left superior cerebellar peduncle. Multiple flow voids were demonstrated within the tumor. The diagnosis of a hemangioblastoma is highly suspected.

**1:17** The hydrocephalus was also noticed from the enlarged ventricles. After admission, a ventricular drainage with a subcutaneous Ommaya reservoir implantation was performed to relieve the hydrocephalus and normalize the intracranial pressure. This technique can be useful for further brain relaxation during the surgery, and as a fail-safe as the postoperative cerebellum or brainstem edema might cause hydrocephalus aggravation.

**1:45** Preoperative angiography confirmed the blood supply of the tumor came mainly from the left superior cerebellar artery. An Echelon-10 microcatheter was plugged into the tumor in a wedge pattern due to the small diameter of the feeders, which occluded the path of the reflux completely. Onyx-18 was used for embolization as it can be observed during the open operation, as a label of supplying arteries. Three milliliters of Onyx-18 was injected and the postembolization angio demonstrated a total occlusion of those feeders. The cast matched 90% to the configuration of tumor.

**2:25** The patient was put in a left park-bench position with the head turned 15° away from the sagittal plane, and the tumor was approached via a left occipital interhemispheric transtentorial approach. This surgical trajectory was also confirmed by the neuronavigation. Compared with the surgical exposure via a supracerebellar infratentorial approach, the larger area of mesencephalic-cerebellar junction exposed by the occipital interhemispheric transtentorial approach provides the surgeon with more comfortable maneuverability and better control of the vasculatures, and it also allows less cerebellar retraction or transgression and a more direct surgical trajectory.

**3:08** The left paramedian occipital craniotomy was performed across the superior sagittal sinus to maximize the exposure to the midline structures after tack-up sutures. The left occipital lobe was retracted laterally as the drainage from the ventricles had relaxed the brain tissue. The tentorium was incised parallel to the straight sinus, and a small piece of tentorium was resected to expose the cerebellum. The tumor was embedded underneath a thin layer of cerebellar parenchyma, which was transgressed to expose the tumor. The upper portion of the tumor was adhesive to the arachnoid membrane and deep veins around the pineal region. It could be sharply dissected with microscissors after pushing the tumor caudally. Then, the main feeding artery, left superior cerebellar artery, was visualized at the beginning of tumor resection. The Onyx can be seen inside the SCA feeders supplying the tumor, which made the SCA conspicuously identifiable and its normal trunk could be well protected during the operation. It could be controlled easily and at the early stage, which is one of the major advantages compared with the supracerebellar infratentorial approach in this case.

**4:23** The tumor is adhesive to the midbrain. We found the fourth nerve can be a very useful landmark to keep the dissection within the midbrain parenchyma and on the correct dissection plane on the pia surface. We performed sharp dissection along the SCA and the pia surface of the midbrain. However, the caudal part of tumor invaded into the cerebellar parenchyma; we had to separate it in a subpial fashion. After the lateral and bottom sides of the tumor was dissected free, we turned to deal with the medial tumor surface. A hemangioblastoma should be treated like an AVM. Numerous feeders from the pia and the distal SCA fed the tumor, and the tumor should be dissected in an en bloc fashion. Bleeding from those feeders was easy to control because the embolization had decreased the intratumoral arterial pressure. The left SCA was skeletonized while all other small feeders were cauterized. The rest of the tumor was separated from the cerebellum in various directions with limited bleeding. The tumor must be totally dissected from surrounding parenchyma, leaving the ligation of the draining veins as the final step. After the total removal of tumor, an aneurysm clipper was applied on the cutting end of the main feeding trunk to secure the occlusion due to potential recanalization from the Onyx embolization. The estimated blood loss was 300 ml; no blood was needed. No motor evoked potential or sensory evoked potential decrement was encountered during the operation.

**6:05** The patient was alert postoperatively without any new neurological defect. Dizziness aggravated transiently but gradually recovered to his preoperative baseline in 1 week. His Glasgow Outcome Scale was 4 at discharge because he still needed ambulatory assistance due to the ataxia. The pathological findings confirmed the diagnosis of a hemangioblastoma. We believe this case can demonstrate the safety and efficacy of the hybrid treatment for a mesencephalic-cerebellar hemangioblastoma.
